# Donald A. B. Lindberg, MD, Honorary MLA Member

**DOI:** 10.5195/jmla.2020.878

**Published:** 2020-04-01

**Authors:** Betsy L. Humphreys, Megan Rosenbloom

**Affiliations:** Arlington, VA, betsyhumphreys@verizon.net; Obituaries Editor, *Journal of the Medical Library Association,* and Norris Medical Library, University of Southern California, Los Angeles, CA, megan.rosenbloom@usc.edu

## Abstract

Donald Allan Bror Lindberg, MD, director emeritus of the US National Library of Medicine (NLM) and Honorary MLA Member, died on August 17, 2019, in Bethesda, Maryland. Lindberg was NLM’s longest serving director and led the library through extraordinary changes that affected health sciences libraries and access to health information worldwide.

Donald Allan Bror Lindberg, MD, director emeritus of the US National Library of Medicine (NLM) and Honorary Member of the Medical Library Association (MLA), was born on September 21, 1933, in Brooklyn, New York, and died on August 17, 2019, in Bethesda, Maryland. Lindberg was NLM’s longest serving director, leading the library from 1984 to 2015 through extraordinary changes that affected health sciences libraries and access to health information worldwide.

Lindberg earned a bachelor’s degree in biology from Amherst College in 1954, and his medical doctor (MD) degree from the College of Physicians and Surgeons, Columbia University, in 1958. He married Mary Musick, a nurse he met at Columbian Presbyterian Medical Center (CPMC), in 1957 while in medical school. Lindberg interned in pathology at CPMC. In 1960, he went to the University of Missouri School of Medicine as a resident in pathology and director of the Medical Center Diagnostic Microbiology Laboratory. A pioneer in computer-based clinical laboratory systems, the use of large data sets to establish normal laboratory values, and expert systems, he also directed statewide information systems, established an NLM-funded graduate medical information sciences training program, and chaired a department in the School of Library and Information Science. When he left Missouri in 1984, Lindberg was a professor of pathology and of information science, an international leader in the field that would become known as biomedical informatics, and superbly qualified to lead NLM.

**Figure:**
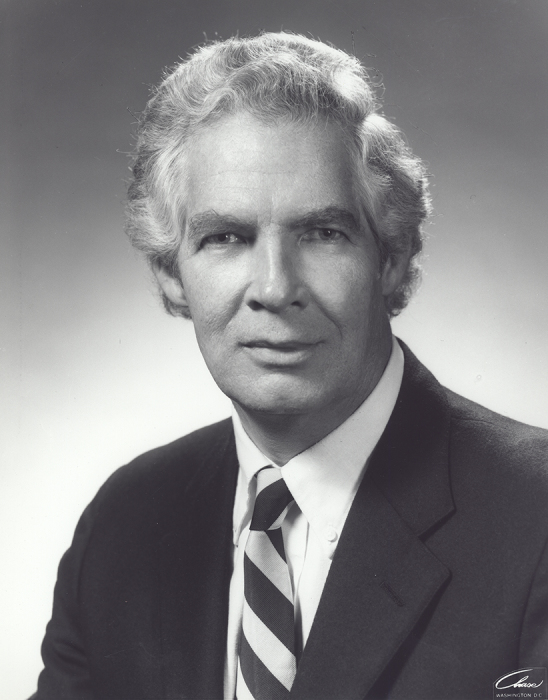


NLM’s basic mission, “to assist the advancement of medical and related sciences and to aid the dissemination and exchange of…information important to the progress of medicine and to the public health” [1], remained the same throughout Lindberg’s tenure, but the way NLM pursued it broadened and changed dramatically. When Lindberg arrived in Bethesda in 1984, NLM served health professionals and scientists primarily through libraries and did not attempt to serve the general public. Librarians were the principal direct users of NLM systems; end user searching was only just emerging. By his retirement in 2015, librarians were a small subset of NLM’s millions of direct users, who included scientists, health professionals, the general public, and computer programs worldwide. Together, these users conducted billions of searches of NLM’s richly linked bibliographic data, born digital and digitized full text, consumer health information, chemical and toxicological data, genomic and genetic variation data for humans and pathogens, clinical trials data, images, and standard terminologies.

NLM resources became critical infrastructure for biomedical and informatics research, education, electronic health records, health care, detection of and response to epidemics, and many commercial products and services. The National Network of Libraries of Medicine (NNLM) included public libraries and community information centers in addition to health sciences libraries, reflecting vastly expanded outreach efforts to increase awareness and use of NLM resources by the public and health professionals, with an emphasis on minority and disadvantaged populations [2]. NLM’s role in this health information revolution was foreseen by Lindberg in his preface to the 1987 NLM Long Range Plan, “What seems needed now is to adapt these general and useful technologies to the specific jobs of biomedicine,” Lindberg wrote. “Progress might eventually come in any case, but a concerted effort on the part of the National Library of Medicine could speed this up” [3].

An early spectacular example of Lindberg’s success in this mission was the 1988 legislation to establish the National Center for Biotechnology Information (NCBI) at NLM. This was the first of seven congressional extensions to NLM’s responsibilities during Lindberg’s tenure, all reflecting confidence in his leadership and NLM staff and advocacy by NLM supporters, including MLA, the Association of Academic Health Sciences Libraries (AAHSL), and the Friends of the NLM, established in 1986.

Lindberg and his wife Mary attended MLA meetings regularly, where he presented the NLM update with appealing visuals and was a great sport at the social events. Diane McKenzie, FMLA, remembered he relished his role as the crowing rooster during a “Comin’ Round the Mountain” game at the 1986 Pacific Northwest and Midwest Chapters conference in Jackson Hole, Wyoming. “He did it with gusto,” she recalled. “He earned my eternal support with his willingness to be silly with the rest of us” [4].

He sought intelligence from the field about challenges faced by NLM’s users and innovative but sensible ideas for improving information access. He could be blunt about ideas that he considered foolish, but persistence and data could change his mind. Lindberg advanced projects to modernize NLM systems and establish a preservation program. His personal stamp was evident in the decision to make DOCLINE use free to libraries and in the promotion of end user searching via the Grateful Med PC-based search package, a move that was unpopular with many librarians at the time. In contrast, his prescient decision not to produce MEDLINE CD-ROM products—opting instead to facilitate their commercial development and evaluation in libraries across the country—pleased the information industry and librarians alike, as did his campaigns to increase the number of journals indexed and the use of permanent, acid-free paper in biomedical journals.

In the 1990s, Lindberg’s concurrent appointment as first director of the National Coordination Office of the multi-agency High Performance Computing and Communications (HPCC) Initiative in the Executive Office of the US President led to expanded support for Internet connections for libraries and hospitals [5] and NLM’s early embrace of the web browser as the interface to its systems.

The spread of the Internet and masterful handling of related political issues, helped by MLA and AAHSL, allowed NLM to make MEDLINE free via PubMed in 1997. This revolutionary change had immense benefits worldwide, although PubMed had some drawbacks for skilled librarian searchers. Use of MEDLINE/PubMed increased exponentially and set the stage for development of MedlinePlus and other consumer health services as well as innovations in outreach to health professionals and the general public via NNLM and other partners, including the Historically Black Colleges and Universities. Free MEDLINE on PubMed also encouraged scientists to lobby for free access to full text, starting a process that would lead to PubMed Central, Clinicaltrials.gov, and eventually federal requirements for free public access to research results. The latter would encourage health sciences librarians to develop new roles in research compliance and data management.

Lindberg encouraged audiovisual and imaging research at NLM and its application to the library’s visually rich historical collections. The web’s multimedia capabilities supported broad access to these treasures and to NLM’s historical exhibitions, which were greatly expanded with his direct involvement in the mid-1990s. Traveling versions of NLM exhibitions became important tools for NNLM outreach and intensive efforts to attract diverse young people to health sciences careers. Lindberg traveled to Alaska, Hawaii, and many other states to conduct the interviews that formed the core of *Native Voices: Native Peoples’ Concepts of Health and Illness*, a demonstration of respect that was essential to obtaining them. “His sincere interest in learning about and sharing the lessons about Native health therapies and beliefs was very moving,” recalled Ruth Holst, AHIP, FMLA, former MLA president. “I would never have spent as much time with or learned as much about these indigenous peoples if he had not been so instrumental in drawing attention to them” [6].

Under his leadership, virtually every expansion in NLM programs—including but not limited to medical informatics; the Unified Medical Language System (UMLS); the establishment of NCBI; HPCC involvement; HIV/AIDS, public health, consumer health, and disaster preparedness information; public access; and clinical trials—led to new leadership appointments for NLM librarians, new funding for librarian-directed research and demonstration projects, and new training and career development opportunities for librarians. “The relationship developed into a mutually supportive one where we encouraged NLM to make access to the databases free to the public and NLM supported a number of MLA initiatives through funding including minority recruitment into the profession, health information literacy, hospital librarianship, and the education and training for health sciences librarians,” said former MLA Executive Director Carla J. Funk [7].

Lindberg mentored many librarians at NLM and elsewhere, including participants in the Woods Hole (later, Georgia) medical informatics course, NLM Associate Fellows, and NLM-AAHSL Leadership Fellows and Mentors. Like librarians on NLM’s staff, advisory committees, and planning panels, participants in these programs had an opportunity to appreciate his very broad interests in the humanities as well as science and to enjoy his sense of humor, integrity, warmth, and genuine interest in their careers and well-being. Among the many former MLA presidents who knew and respected him, J. Michael Homan, AHIP, FMLA, remembered Lindberg as a “trustworthy, brilliant, and effective ambassador for NLM and NIH” [8], and Beverly Murphy, AHIP, FMLA, viewed him as “a great man whose contributions will stand the test of time” [9].

Lindberg received many honors during a career that, as the NLM Board of Regents stated in 2015, “changed fundamentally the way biomedical knowledge and health information is collected, organized, and made available for public use” [10]. MLA’s Donald A. B. Lindberg Research Fellowship is one of the awards, professorships, and lectures named for him.

Donald Allan Bror Lindberg, MD, is survived by his wife Mary Musick, two of their three sons, two grandchildren, and his brother.

**Betsy L. Humphreys, FMLA**, betsyhumphreys@verizon.net, Arlington, VA

**Megan Rosenbloom, AHIP**, megan.rosenbloom@usc.edu, Obituaries Editor, *Journal of the Medical Library Association*, and Norris Medical Library, University of Southern California, Los Angeles, CA
